# Women’s experiences of transfer from primary maternity unit to tertiary hospital in New Zealand: part of the prospective cohort Evaluating Maternity Units study

**DOI:** 10.1186/s12884-015-0770-2

**Published:** 2015-12-18

**Authors:** Celia P. Grigg, Sally K. Tracy, Virginia Schmied, Amy Monk, Mark B. Tracy

**Affiliations:** University of Sydney, Sydney, NSW Australia

**Keywords:** Birthplace, Transfer, Women’s experiences, Primary maternity unit, Tertiary hospital, Continuity of care, Place of birth

## Abstract

**Background:**

There is worldwide debate regarding the appropriateness and safety of different birthplaces for well women. The Evaluating Maternity Units (EMU) study’s primary objective was to compare clinical outcomes for well women intending to give birth in either a tertiary level maternity hospital or a freestanding primary level maternity unit. Little is known about how women experience having to change their birthplace plans during the antenatal period or before admission to a primary unit, or transfer following admission. This paper describes and explores women’s experience of these changes-a secondary aim of the EMU study.

**Methods:**

This paper utilised the six week postpartum survey data, from the 174 women from the primary unit cohort affected by birthplace plan change or transfer (response rate 73 %). Data were analysed using descriptive statistics and thematic analysis. The study was undertaken in Christchurch, New Zealand, which has an obstetric-led tertiary maternity hospital and four freestanding midwife-led primary maternity units (2010–2012). The 702 study participants were well, pregnant women booked to give birth in one of these facilities, all of whom received continuity of midwifery care, regardless of their intended or actual birthplace.

**Results:**

Of the women who had to change their planned place of birth or transfer the greatest proportion of women rated themselves on a Likert scale as unbothered by the move (38.6 %); 8.8 % were ‘very unhappy’ and 7.6 % ‘very happy’ (quantitative analysis). Four themes were identified, using thematic analysis, from the open ended survey responses of those who experienced transfer: ‘not to plan’, control, communication and ‘my midwife’. An interplay between the themes created a cumulatively positive or negative effect on their experience. Women’s experience of transfer in labour was generally positive, and none expressed stress or trauma with transfer.

**Conclusions:**

The women knew of the potential for change or transfer, although it was not wanted or planned. When they maintained a sense control, experienced effective communication with caregivers, and support and information from their midwife, the transfer did not appear to be experienced negatively. The model of continuity of midwifery care in New Zealand appeared to mitigate the negative aspects of women’s experience of transfer and facilitate positive birth experiences.

## Background

Little is known about the experience of women who find themselves having to change their planned place of birth or transfer from primary maternity units to tertiary hospitals during labour. Transfer is one of the issues taken into consideration by those planning to give birth in a freestanding primary unit [1]. Transfer has the potential to impact on the physical and/or emotional wellbeing of those involved and on social perception of primary units as safe birthplaces [2–4].

Contemporary research on women’s experience of transfer from primary units to hospital is limited, with the Birthplace in England project the most comprehensive and current available, although it reports labour transfers only and combines data from both onsite and freestanding unit transfers [2]. Two other older British studies [3, 4] and one contemporary Danish study [5] report on women’s experiences of antenatal and labour transfers from freestanding units. Almost all of the identified transfer literature, involved transfer to a different model of care and caregiver for most or all women; which might involve the woman changing primary caregiver from an independent midwife or primary unit employed midwife to a hospital employed midwife in the obstetric-led hospital. One recent New Zealand study, in the context of continuity of care, explored transfer from a rural perspective [6].

More broadly, there is research into aspects of women’s feelings or experience of transfers from planned home birth to hospital [7–11], or transfers from primary to secondary care [10, 12]. There is also a larger contemporary body of work on the wider topic of women’s birth experiences and factors found to be influential [13–17].

In New Zealand in 2010 85.4 % of births occurred in a secondary or tertiary hospital, 10.8 % in a freestanding primary unit, 3.2 % at home and 0.6 % at an unknown location [18]. A TMH has specialist obstetric, anaesthetic and paediatric staff and facilities on site and available at all times. A PMU has midwifery services on site and available at all times, but no medical staff or specialist facilities. In many areas women do not have the option of giving birth in a PMU birth, following the centralisation of maternity hospitals which began in the 1920’s [18, 19]. All PMUs in New Zealand are freestanding and many are rural. The Christchurch area has one TMH and four midwife-led PMUs, with two of the PMUs in the rural hinterland, not the city itself.

The New Zealand maternity system has continuity of care as a core tenet [20] resulting in women receiving continuity of care regardless of planned or actual birthplace. Women choose their own ‘lead maternity carer’ (LMC) who continues to provide primary level care throughout her maternity experience-antenatal, labour/birth and six week postpartum. Most LMCs are community based midwives [21]. The midwife generally remains the primary caregiver even if complications arise, requiring obstetric consultation and a change of plan antenatally or a transfer between facilities during labour and birth [22]. (For a comprehensive description of New Zealand’s unique maternity system see Grigg & Tracy 2013 [23].)

This paper reports on the Evaluating Maternity Units (EMU) study which is the New Zealand arm of an Australasian prospective cohort study. The primary aim of the overall study is to compare the clinical outcomes for well (‘low risk’) women, intending to give birth in either an obstetric-led tertiary level maternity hospital (TMH) or a freestanding midwifery-led primary level maternity unit (PMU) in Australia or New Zealand. The Australian clinical outcomes have been reported previously [24]. The New Zealand arm of the prospective cohort study is a mixed methods design and one of its aims is to describe and explore women’s birthplace decision-making [1, 25]. The first paper from the EMU study on the subject of having to change from a chosen place of birth, reported on the timing, frequency, reasons, urgency and outcomes of transfer [26]. This paper describes women’s experiences of having to change the place of birth during the antenatal time or before admission in labour; or having to transfer in labour or postnatally from PMU to a TMH in New Zealand.

## Methods

A mixed method methodology was chosen for the New Zealand arm of the Evaluating Maternity Units project, to address the complexity of issues around birthplace and optimise the opportunity the study provided to collect clinical outcome data and hear and give voice to women’s experiences. While a cohort study is traditionally a quantitative research, the inclusion and integration of qualitative textual data and analysis renders the research mixed methods. This required the adoption of a mixed method methodology which informed the study strategy. It was grounded in a pragmatic approach [27–29], with a ‘concurrent QUANTITATIVE (QUAN) + qualitative (qual)’ typology [30, 31]. Mixed methods research uses capital letters to indicate the dominant data source, and the abbreviations of ‘quan’ representing quantitative data and ‘qual’ depicting qualitative data.

Quantitative data were analysed using descriptive statistics and the qualitative data were analysed using thematic analysis. The data were integrated for the interpretation stage, and the findings were also integrated through inferences made in the discussion [31]. Ethics approval was granted by the Upper South B Regional Ethics Committee (URB/09/12/063).

### Sample and recruitment

All women booked to give birth in one of the four primary maternity units during the recruitment period were invited to participate. Women who booked into the tertiary hospital during the same period, and were well pregnant women (at ‘low risk’ of pregnancy complications), were also invited to join to study. (The hospital booking forms were the means of identifying eligible women.) For the purposes of this study, ‘low risk’ was defined as not having any level two or three referral criteria as defined in the New Zealand Referral Guidelines (2007) [32]. For example, women who had had a previous caesarean section or were expecting twins were ineligible. Recruitment was undertaken by the lead author. Eligible women were sent a postal invitation to join the study, with a follow-up phone call to those who did not respond. Additionally, some women were invited by their midwife (using the study invitation brochure, information sheet and consent form). Those women who returned signed consent forms were entered into the study. Consent to join the study included consent to receive two surveys, via post or online, one at six weeks and another at six months postpartum. Recruitment began in March 2010, was suspended for one month after a major earthquake in September 2010, and stopped prematurely after a subsequent severe earthquake in February 2011. Following the September earthquake two primary units were closed for a week and services had resumed within two weeks. After the February earthquake the city’s busiest PMU closed and was subsequently demolished, and another was closed for 7 weeks. The semi-rural PMUs remained open. Participants’ births occurred between March 2010 and August 2011. More details regarding the study’s sample and recruitment have been reported in detail previously [1, 26]. All of the participants in this study had a midwife LMC, and all remained in their care except one, whose rural midwife handed over care to TMH midwives after transfer in labour. Consequently, the potential confounding impact of having two different models of care for women in the two cohorts, was minimised. This allowed for the comparison of the differences in outcomes related to planned birthplace independently of midwifery model of care.

### Data collection

Data collection for all phases of this study, including the survey and its construction, have been described in detail previously [1]. While three types of data were collected in the EMU study only one dataset was used for this article–that from the six week postpartum survey, which included closed ended questions (quantitative) and the (qualitative) open ended questions. The survey aimed to provide a comprehensive coverage of women’s birthplace decision-making; pregnancy, labour and postnatal experience and care, and the wellbeing of themselves and their baby at six weeks postpartum. It comprised nine pages and 51 questions, some of which had multiple sub-questions [1]. The majority of questions were ‘closed’ (tick box or Likert scale), with 13 questions open ended and nine of those sought explanatory or descriptive detail. Questions covered several topics, including:

- women’s birthplace decision-making
- several aspects of their antenatal, labour and postnatal experiences and care
- their feelings and worries regarding labour and birth
- where their baby was born
- details of any antenatal change of plan or transfer in labour and how they felt about it
- their antenatal plans for feeding their baby
- details of feeding method (s) up to the time of completing the survey, and
- details of any health problems they or their baby experienced in the first six weeks.

The survey was sent via post, unless participants chose to receive it online by giving their email address on the study consent form (60 %). The surveys received via post were entered into the online format (Survey Gizmo) by a contracted data entry operator. Data entry accuracy was checked by CG, with a random sample of 10 % of the surveys checked and found to be 100 % accurate. Survey responses analysed for this paper included the ‘closed’ and ‘open’ questions. The ‘closed’ Likert scale (quantitative) questions analysed were

- How did you feel about the decision to give birth elsewhere? Scale options: Very unhappy, Unhappy, It didn’t bother me at all, Happy, and Very happy.
- If you transferred from one hospital to another during labour/birth, how was this experience for you? Scale options: Very Good, Good, Neutral, Bad, and Very Bad.

The open-ended (qualitative) questions analysed for affected participants were:

- What did you like most about the care provided for this pregnancy, labour/birth and first six weeks after birth?
- What, if anything, did you not like about the care provided for this pregnancy, labour/birth and first six weeks after birth?
- Is there anything else you would like to tell us?

### Data analysis

The survey responses were initially downloaded from the online survey provider (Survey Gizmo) in SPSS software. The qualitative survey data were manually reviewed by CG and inductively grouped and coded, with themes identified, using thematic analysis [33]. The themes were identified by examining commonalities, relationships and differences across the dataset and the identified patterns are reported as themes [34]. The coding and interpretation was checked for representation and consistency by ST. An audit trail was kept linking the raw data and themes. The numerical ‘study code’ identifier is used for quotes. The open-ended responses were managed with NVivo (version 10.0), which provided an audit trail linking the raw data and themes. Quantitative data were analysed using descriptive statistics (SPSS version 22). Following initial analysis, the quantitative and qualitative survey data were combined and assessed for complimentarity or dissonance. Women were identified as being in either the PMU or TMH cohort, with their four digit unique identifier used in quotes.

## Results

### Participants

**Fig. 1:**
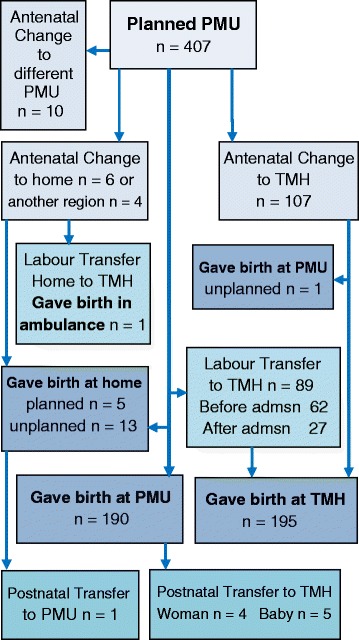
Flow chart of PMU changes, transfers and birthplaces

A total of 407 study participants planned to give birth in a PMU on entry into the study. Table 1 shows the demographics of the women who responded to the six week postpartum survey. Of the 238 women in the PMU cohort who experienced any type of birthplace change of plan or transfer 174 responded to the survey questions relating to transfer, representing a response rate of 73 %. The data presented here are from these women’s responses-96 who changed antenatally, 55 who changed prior to admission in labour and 21 who transferred after admission to a PMU, and 2 who transferred postnatally. Sixty four of these women responded to one or more of the three open ended questions (above). Figure 1 details the birthplace plan changes, transfers and eventual birthplace for the PMU cohort. (Details of the whole study recruitment, inclusions and exclusions details have been reported previously [26].

**Table 1:** Survey respondents’ demographics

Demographic	PMU (%)	TMH (%)	*P* value
	*n* = 330	*n* = 228	(Chi-Square 95 % CI)
Parity			.001
0	41.6	53.3	
1	36.7	37.0	
2-4	20.9	9.3	
≥5	0.9	0.4	
Age			.083
<25	11.3	7.3	
25-29	33.2	25.6	
30-34	40.9	48.3	
35-39	12.8	15.8	
≥40	1.5	3.0	
Ethnicity			.365
NZ European	76.0	78.2	
Māori	5.6	2.6	
Other	18.1	18.8	
Partner			.748
Yes	91.6	91.1	
No	7.6	8.2	
Education			.335
No post-school completed	20.2	15.7	
Apprenticeship, certificate	16.6	13.9	
diploma	16.9	17.8	
degree	46.2	52.6	
Income			.001
< $25,000 pa before tax	6.1	6.2	
$25,001–$50,000	29.1	15.0	
$50,001–$75,000	30.4	31.0	
>NZ$75,000	34.4	47.8

### Results of closed survey questions

#### Antenatal birthplace plan changes

Of the 127 PMU women who changed birthplace plans antenatally 96 (72 %) of them answered the question regarding how they felt about the decision to give birth elsewhere. Table 2 details the responses to the Likert scale survey question. Of the five options the one with the greatest response, for those who changed their planned birthplace antenatally. was ‘it did not bother me at all’, with one third of respondents selecting it.

**Table 2:** How women felt about the decision to give birth elsewhere

Time frame of decision to change birth place	Very unhappy	Unhappy	It didn’t bother me at all	Happy	Very happy
	*n* (%)	*n* (%)	*n* (%)	*n* (%)	*n* (%)
Antenatal (*n* = 96)	13 (14)	28 (29)	32 (33)	16 (17)	7 (7)
Labour prior to admission (*n* = 54)	2 (4)	16 (30)	23 (43)	7 (13)	6 (11)
Labour after PMU admission (*n* = 21)	0	8 (38)	11 (52)	2 (10)	0

### Labour change of plan before admission to the PMU

Of the 76 women who changed birthplace plans in labour and prior to admission to the PMU 54 (71 %) responded to the survey question rating how they felt about the decision to give birth somewhere other than where they had planned, as detailed in Table 2. Again more women were unbothered by the change of plan than were either unhappy or happy about it, with 43 % selecting this option.

### Labour transfer (post-admission) from PMU to TMH

The survey question regarding how women felt about the decision to give birth somewhere other than they had planned was answered by 21 (78 %) of the 27 women who transferred after PMU admission. As detailed in Table 2 none rated themselves as either ‘very unhappy’ or ‘very happy’. and the majority were unbothered by the decision to transfer. The survey asked those who transferred from one hospital to another during their labour/birth how the experience was for them. Nineteen of the 27 women (70 %) who transferred from the PMU responded to the question regarding their experience of transfer, see Table 3.

**Table 3:** How women who transferred between facilities during labour/birth felt about the experience

Experience of transfer	Very Bad	Bad	Neutral	Good	Very Good
		*n* (%)	*n* (%)	*n* (%)	*n* (%)
Women (*n* = 19)	0	5 (26)	6 (32)	5 (26)	3 (16)

In summary, the greatest proportion of women who responded to the question asking how they felt about the decision to change their planned birthplace (antenatally or in labour) rated themselves as feeling ‘neutral’ (66 women-38.6 %). Only 15 (8.8 %) of the survey respondents rated themselves as feeling ‘very unhappy’, and 13 (7.6 %) were ‘very happy’ about the decision. Women who transferred between facilities during labour and birth were asked to rate their experience of the transfer, 8 (42 %) of respondents found the experience of transfer ‘good’ or ‘very good’, 6 (32 %) ‘neutral’ and 5 (26 %) ‘bad’; none rated it ‘very bad’. (Of note are the small numbers involved.) With 38 % of those who transferred rating themselves as ‘unhappy’ about the *decision* to transfer but only 26 % rating the *experience* of transfer as ‘bad’; and only 10 % rating themselves ‘happy’ about the *decision* but 42 % rating the *experience* ‘good’ or ‘very good’, it appears that the women were more unhappy about the decision to transfer than the experience of transfer itself. Only the women who transferred between facilities were asked about their experience of transfer. For those who changed their plan from the community either antenatally or in labour prior to admission to the facility, there was no physical ‘transfer’, only a transfer of intent. There was also no transfer of caregiver or model of care for the participants who changed their planned birthplace antenatally or in early labour, or those who transferred between facilities after admission in labour.

### Findings from open-ended questions

Four themes were identified from the responses of those who experienced a change of plan either antenatally or before admission in labour or having to transfer from the PMU in labour or postnatally: ‘not to plan’, control, communication and ‘my midwife’. There was interplay between the themes, with women’s experience of ‘not to plan’ being impacted by the other factors, which appeared to cumulatively affect the overall experience. (The quotes included below are coded as “liked”, “disliked” or “general” to reflect which open-ended question is being answered).

### Not to plan

The birth not going ‘to plan’ was the overarching theme identified. For some the birth was ‘not to plan but okay’ and others it was ‘not to plan and not okay’. For example, a woman who changed plan antenatally on clinical indication, her birth was ‘not to plan but okay’:

[general]: “*while i would have loved to birth at* [*PMU*] *it was safer for me to be at* [*TMH*]. *luckily i managed a natural vaginal birth with no major issues apart from bleeding in labour* (*bubs was fine during though*). *i had an amazing birth experience*” (3414).

One of the few women who moved out of the region following the earthquakes also seemed to find that while her experience wasn’t to plan it was still okay:

[disliked]: “*the upheaval of the Feb earthquake*, *very nervous having to move cities at 35 weeks pregnant and have a stranger arrange your birthing plans*, *but* [*the hospital staff in another region*] *were great*” (3517).

For a few who changed plan antenatally their birth was not to plan and not okay. For example:

[disliked]: “*Didn*'*t like the fact I was told I was unable to birth at* [*PMU*] (*although I understand the reasons*). *and hated the fact I felt like I was tied to the bed with monitors and needles during the birth*” (3262).

The same sentiments were expressed by some of the women who changed plan prior to admission in labour:

[general]: “*Still felt like I wasn*’*t fully sure why I had a C*-*section. Just wasn*’*t the experience I had hoped for*–*really didn*’*t want a C*-*section*–*I felt induction done to cover their backs* (*1* % *chance of infection*…) *felt like things could have been different*” (3037);

[general]: “*I was annoyed I had to go to* [*TMH*], *it was super*-*short notice and I was panicking*, *I do realise I had to go there but just wasn*’*t what I*’*d planned to do*” (4078).

Of the women who transferred from a PMU to the TMH in labour none expressed sentiment of ‘not to plan and not okay’; those who commented on their birth not going to plan appeared to have experienced transfer without trauma:

[disliked]: “*I didn*'*t have any real issues apart from my birth not going quite to plan*”, [general]: “*I had a very positive birthing experience and i am loving motherhood*” (3548); [general]: “*even though the outcome of my labour was not how I thought it would be i was never upset or scared*…” (3237).

### Control

For some women the perceived sense of ‘control’, was an issue and was identified as a theme. Their capacity to be in control of decision-making was important for some women. The theme applied in each context of change/transfer–antenatal, labour (pre-admission and post-admission) and postnatal. Loss of control was experienced by some women. For example, one woman, whose antenatal plan changed to give birth in another region due to earthquakes, commented that:

“*the loss of control in labour with Drs in particular not explaining procedures properly and taking decision*-*making away in a time when I felt too vulnerable to properly fight for it*-*esp. after the quake*” (3367).

Another, whose plan changed antenatally due to a clinical reason, commented that:

[general]: “*My birth experience was very far from what i had imagined*, *and although I tried not to be disappointed and thankful that I now have a beautiful*, *healthy baby boy. I think that mentally the stress and lack of control and the number of staff* (*all very lovely i want to add*) *that I encountered during my labour* (*I*'*m guessing around 30 people*) *was very distressing for me*… *it wasn*'*t the pain that I found hard*-*just the hectic nature and lack of control i had throughout the whole process*” (3082).

One woman who had a pre-admission labour plan change experienced stress and difficulty maintaining control (she wrote 750 words expressing her anger about her experience at the TMH), which started with:

[disliked]: “*the attitude of the attending doctors*… *their clear preference was to administer syntocinon to* ‘*get things going so I could have my baby faster*’ *as they* ‘*had a quiet night in the hospital*’. *I had to argue for my natural birth*, *making the case that I was not a medical emergency and it was perfectly valid to* ‘*wait and see*’. *I had to ASK how long we could safely* ‘*wait and see*’… *and when told* ‘*17 h*’ *I said* ‘*in that case that was how long I wanted to give my body to start labour*’, *I was still told* ‘*well how about we not put a specific time on it and lets re*-*evaluate in the morning*’ *despite having already made it clear that I wanted to wait as long as I safely could*…” (4065).

In contrast, the positive impact of maintaining control is illustrated by the following comment from a woman, who changed to plan a home birth antenatally:

“[*liked*] *I had control of my own labour and delivery and feel proud that I could have a natural birth after two previous inductions*” (3513).

Similarly, one woman who transferred after PMU admission illustrated her experience of maintaining her sense of control and decision-making power:

[liked]: “*The support of our fantastic midwife and how my views on labour were supported wholeheartedly by her*…”, [general] “…*To transfer was necessary but I did get offered the chance after two hours of pushing and decided against it*, *really wanting to birth at* [*PMU*], *however the next time I got offered I decided for the health of my child I needed to go*” (3359).

The maintenance or loss of a sense of control expressed by the women appeared to influence the sense of positivity or negativity of the experience which, for some, explicitly included decision-making power.

### Communication

Communication, whether positive or negative, was the third theme identified. The way that the staff at the facility (PMU and/or TMH) or others, such as ambulance drivers, communicated with the women affected the women’s experience. Negative communication was experienced by some; for example, a woman who changed plan antenatally on clinical indication commented:

[disliked]: “*The fact I had an emergency c*-*section under GA so missed my baby*'*s birth. I haven*'*t had my questions answered about what happened as my baby was sick after birth and I don*'*t know why or what happened*”, [liked]: “*My backup midwife and the care and support she has provided for me and my baby*” (4091).

Another woman, who changed plan prior to admission in labour, experienced poor communication with health professionals:

[disliked]: “… *the near*-*total consistency with which all staff coming into our room failed to acknowledge my husband and addressed only me. This had the effect that my husband felt like a completely marginalised piece of furniture*… *This in turn had the effect of making us both feel depressed*” (4065).

In contrast, positive communication influenced other women’s experience. For example, a woman who had a clinical indication to change plans antenatally:

[liked]: “*My concerns were taken seriously and when complications occurred they were dealt with quickly and i was given the care that i needed for my condition and post*-*partum complication*”, [disliked]: “*nothing*-*my LMC and her back up did a wonderful job*” (3344).

Another woman, who was transferred from a PMU, also experienced communication positively:

[general]: “*Even though the outcome of my labour was not how I thought it would be i was never upset or scared as i always felt that i was being told everything that was going on and was well informed about everything before i was asked to make any decisions*” (3237);

### My Midwife

‘My midwife’ was the fourth theme identified. Given the context of continuity of care in New Zealand, all 692 of the study participants had ‘their own’ midwife (who had one or two back-up midwives), who provided her primary maternity care regardless of the birthplace, plan change or transfer. (The only exceptions were the five who left the region and one whose rural midwife handed over care to TMH midwives after transfer.) The women identified their midwife, and the relationship they shared, as an important part of their experience. A few women experienced lack of care or support from, or poor communication with, their midwife which had a negative impact on them:

[disliked]: “*I felt we did not communicate very well with the midwife once complications began and this caused a lot of confusion for myself and my support people* (*my husband and my mum*).” (4089); and

[disliked]: “*My midwife was totally unsupportive and disinterested during the labour*… *She didn*'*t give any suggestions or advice*, *and was more interested in filling in her paperwork than dealing with me when I was in the final stages of transition. She did NOTHING*! … *It was a very stressful and unpleasant experience*” (4077).

Most of those affected by plan change or transfer identified their midwife as both important and positive in their care:

[liked]: “*my midwife was fantastic and I felt so supported by her and confident that she understood me and what I wanted for all parts of my pregnancy*, *labour*, *birth* etc. *and she did everything she could to make sure that things at hospital in particular were carried out that way*, *especially given that my baby was early*” (*4098*);

[liked]: “*my fantastic midwives who were always so supportive and lovely. They went with all of my decisions and made sure me and my husband were always well informed*” (3548);

[liked]: “*The care given. I had to be transferred to* [*TMH*] *after given birth at* [*PMU*] *as I had a retained placenta. Midwife & student midwife were great*, *and there with me & my husband & new baby right through it all*” (3072).

### Cumulative impact of themes on women’s experiences

The women’s experiences of the three themes–control, communication and their midwife–appeared to influence how they described their overall experience of change in their birthplace plan. Analysis identified that the more themes which were positive about transfer the more positive the woman’s report of her birth experience, regardless of the timing (antenatal, in labour or postnatal) or clinical situation (urgent or non-urgent). Those who experienced a loss of a sense of control, poor or negative communication from care givers, and lack of care, support or information from their midwife seemed to express more negative sentiment, and more often felt that their birth was both ‘not to plan and not okay’. Overall only a few of the respondents described their experience this way, see Table 4 (a). Those who experienced effective support, communication and control described their experience as ‘not to plan but okay’ (Table 4 (b)).

**Table 4:** Exemplars illustrating a cumulative impact of interplay between themes

(a) ‘Not to plan and NOT okay’
[liked]: “*my midwife *- *she finally listened to ME instead of so*-*called professionals*”, [disliked]: “*The hospitals recommendations were unnecessary*/*unwarranted. No attention to my wishes*, *induced for no reason and caused unnecessary pain*/*anguish*/*distress*/*depression*”, [general]: “*as above*, *not happy*!” (antenatal change to TMH for clinical reason, 3212).
[liked]: “*Not much really because i felt i was told nothing about my birth as i ended up having an emergency c*-*section wasn*’*t told why and didn*’*t get to see my baby after he was born for ages*, *also never got told that he wasn*’*t breathing when he was born it was my* [*child health*] *nurse who told me when my baby was over a month old. Very disappointing*” (antenatal change to TMH after earthquake, 3360).
(b) ‘Not to plan and okay’
[liked]: “*I never once felt worried*, *even when things were not going so well in Labour and after*, *I never had any reason to feel that everything would not be fine in the end. NICU*, *the* [*postnatal*] *Maternity Ward at* [*TMH*] *and my midwife were all fantastic and very helpful. Everything was pretty much excellent*, *and when* [*baby*] *decided to come early this posed no problem to anyone*, *the midwife was there straight away and the hospital provided professional fast service*” (pre-admission labour change, 4050).
[liked]: “*My Midwife *- *she was amazing*, *very personable and laid back*”, [general]: “[*TMH*] *had the most wonderful staff*, *from the ambulance drivers to the anesthetist*, *I felt very safe and informed in what was a difficult birth*” (labour transfer PMU to TMH, 3413).
[liked]: “*My son was born with the cord wrapped around his neck twice*, *not breathing*, *limp and very pale. I*’*m thankful to all the staff at both hospitals for acting quickly and saving his life. At* [*PMU*] *I was addressed by my name and felt they had a more genuine interest in me and my son. At* [*TMH*] *I was addressed as mummy and staff communication was very poor*” (postnatal transfer PMU to TMH, 3251).

It is noteworthy that only three women commented on the transfer itself-the quote above regarding the ambulance drivers, one who rated her transfer experience as ‘good’ commented: “*I had to pay for the ambulance*!” (4086), and the other woman, who had a ‘non-emergency’ transfer, wrote notes in the survey margins-“*ambulance took 1 h 20mins to get to us*” and “’*just another case*’, *not at* [*PMU*], *at* [*TMH*]”(4066). Also, a review of all of the responses found none that suggested that any of the respondents were unaware of the possibility of change or transfer when they planned their PMU birth.

### Integration of closed and open survey responses

Integration of the combined closed and open responses data revealed greater complexity in the women’s transfer experiences than the initial independent analysis of the two sets of data. Table 5 illustrates examples of the combined responses, with responses indicative of a pragmatic attitude to the transfer. Most respondents did not want or plan to change birthplace plans. However, no respondents expressed trauma regarding the transfer itself, with none selecting the option ‘very bad’ for the experience of transfer and no comments suggestive of distress and 42 % rating the experience ‘good’ or ‘very good’. The complexity identified included two women who rated their experience of transfer as ‘bad’ yet made comments which suggested that they were not necessarily unhappy about the transfer itself. One of whom was unhappy that the transfer did not occur earlier, and the other seemed unconvinced of the clinical need to transfer and the attitude of TMH staff after transfer (see the final two quotes in Table 5 respectively).

**Table 5:** Combined survey responses from women who transferred from PMU to TMH (in labour)

Experience of transfer rated ‘very good’, Feeling about decision rated ‘unhappy’. (study code 3237)
Reason: “*Long labour*”
Comment: “*Even though the outcome of my labour was not how I thought it would be I was never upset or scared as I always felt that I was being told everything that was going on and was well informed about everything before I was asked to make any decisions*.”
Experience of transfer rated ‘good’, Feeling about decision rated ‘unhappy’ (3359)
Reason: “*After pushing for 4 h & her getting stuck we had an emergency transfer so that suction could be used to help get her out. Was last choice to move but she needed help*.”
Comments:
Liked: “*The support of our fantastic midwife & how my views on labour were supported wholeheartedly by her*…”
General: “*I would not change anything about my labour or care for my next pregnancy*, *apart from hopefully not having to transfer*! *To transfer for me was necessary but I did get offered the chance after two hours of pushing and decided against it*, *really wanting to birth at* [*PMU*] *however the next time I got offered I decided for the health of my child I needed to go*”.
Experience of transfer rated ‘neutral’, Feeling about decision rated ‘unbothered’ (3413)
Reason: “*Started out at* [*PMU*] *was there for about 4 h*, *but the baby failed to progress so was taken to* [*TMH*]”
Comments:
Liked: “*My midwife*–*she was amazing*, *very personable and laid back*”
General: “[*TMH*] *had the most wonderful staff*, *from ambulance drivers to the anesthetist*, *I felt very safe and informed in what was a difficult birth*.”
Experience of transfer rated ‘bad’, Feeling about decision rated ‘unhappy’ (3195)
Reason: “*long latent labour*, *overdue*”
Comments:
Liked: “*that baby was well. home visits*”
Disliked: “*midwife cancelling every second appointment*”
General: “*I was left too long before going to* [*TMH*], *36 h labour*, *meconium*, *cord round neck*, *facing wrong way coming out*, *apgar 3*” [author note: birth >10 h after transfer & obstetric consult]
Experience of transfer rated ‘bad’, Feeling about decision rated ‘unhappy’ (4080)
Reason: “*We had to transfer as per* [*PMU*] *protocol due to meconium in liquor*”
Comments:
Liked: “*my LMC treating as an individual*”
Disliked: “*The obstetricians lack of bedside manner at the birth*”

## Discussion

The study’s qualitative analysis found that a change in birthplace plan was not negatively experienced for women who maintained their sense of control, effective communication with caregivers and felt supported and informed by their midwife, regardless of the timing of the change or the type of birth they experienced. While it was not what they planned or wanted the women knew transfer was a possibility and generally accepted it as appropriate, and most managed the change without too much stress. The stress which was evident was mostly experienced at the tertiary hospital as a result of institutional issues or the approach or manner of those employed there. The Likert scale responses, analysed quantitatively, suggest that the women were more unhappy about the *decision* to change birthplace plan or transfer than the *transfer* itself. The closed Likert-scale responses do not identify what it was that women were ‘happy’ or ‘unhappy’ about, or what was ‘good’ or ‘bad’ about their transfer experience. The women’s narratives provided an opportunity for explanation and facilitated better understanding of respondents’ experiences and perspectives.

A minority of respondents (39.2 %) rated themselves on a Likert scale as unhappy (combined ‘very unhappy’ or ‘unhappy’) about the decision to change birthplace, regardless of when the decision was made. A similar proportion of women were neutral about the decision (38.6 %). The proportion who rated themselves ‘it did not bother me at all’ for change antenatally, pre-admission in labour or post-admission in labour were 34 %, 42 % and 52 % respectively. Despite not wanting or planning to change birthplace overall 22.2 % of respondents rated themselves as ‘happy +/− very’ about the decision.

The themes of control, communication and ‘my midwife’ (interpreted as relational continuity of care) identified in this research are all complex constructs which have been discussed in the context of maternity care previously [11, 13, 15, 35–38]. Defining each of them in the context of birth is difficult and beyond the scope of this paper. They have been identified as contributors to women’s positive birth experiences [5, 13, 15, 39]. Notably the quality of ‘relationship’ is central to each of the themes. The centrality of relationships in the quality of maternity care has also been reported previously [13, 14, 17, 35, 40–42].

‘*Control*’ has been recognised as “a key factor that can enhance or diminish the experience of childbirth” [15]. The EMU study respondents appeared to refer to ‘control’ in terms of self-determination, defined as “the ability to have a birth that is shaped and guided by one’s own inclinations and values rather than those of others” [38]. They referred to losing control to others, usually caregivers, rather than their ‘self-control’. K Cook and C Loomis [10] also found women’s experiences were influenced by the level of control they felt when their birth plan changed–“it is not simply the fact that the birth plan changed that leads to positive or negative feelings, it is… the degree of control that women have over the changes as they are happening” (p.165). Control and participation in decision-making have also been linked to satisfaction with the experience of childbirth previously [14, 16, 36, 43].

‘*Communication*’ with caregivers has the power to be positively or negatively influence women’s experience of transfer and childbirth itself. EMU study participants, who planned a primary unit birth, found supportive and respectful communication with caregivers helped mediate the negative effect of being in a tertiary hospital. The powerful influence of the attitudes and behaviours of caregivers on women’s birth experience has been identified previously [11, 14, 15, 41, 43, 44]. E Ford and S Ayers [44], concluded that the level of support from hospital staff during birth had a greater effect on women’s emotional reactions (particularly perceived control) than stressful events.

The influence of the respondents ‘*midwife*’ on her transfer and birth experience was powerful, whether positively or negatively so. New Zealand women expect to have their ‘own midwife’ (and a back-up midwife), who ‘knows them’ and will provide “individualised care, empathetic understanding and support, and active communication” [45]. Continuity of midwifery care in New Zealand does not refer to ‘teams’ or ‘caseloads’ it refers to ‘one-to-one’-named individuals who contract with a woman to provide her primary maternity care [23]. (Midwives generally work in practices, which provide back-up when it’s needed.) The quality of the relationship is as important as the continuity of care [13, 17, 35, 40–42]. Women in New Zealand have been found to greatly value the type of genuine continuity of care offered: they want a “close and personal relationship with their midwives” [40], one which builds over time as they come to know and trust each other. This system places great importance on the nature and quality of the relationship between the midwife and the woman (and her whanau/’family’), anchored by the concept of partnership [46, 47].

The concept of ‘loss’ was found to be central in earlier transfer research [2, 4, 11], with loss of control and continuity central [4]. Previous research, in a context of no continuity of care following birthplace plan change or transfer, had identified the lack of continuity of care as contributing to women’s sense of ‘loss’ [2–4] or even ‘failure’ [2, 4]. Participants in the current study experienced continuity of care and did not express the sentiments of loss or failure. In contrast to previous research, respondents did not express any sense of ‘disbelief’ or ‘shock’ at an antenatal plan change, as reported by Walker (2000) who described those hearing the news as ‘devastating’ or ‘a bombshell’. Recent Dutch research into women’s experience of transfer from primary to secondary care identified different types of continuity, and found that women valued relational continuity most highly: “management continuity is silver, relational continuity is gold”, although it was not common in that context [12]. The women-centred continuity of care context in New Zealand may have an important role in mitigating the potentially negative impact of birthplace plan changes. Indeed Davis and Walker [48] contend that this unique system, where the midwife woman pair move together fluidly from the community through to secondary or tertiary care and back, helps bridge the ‘normal/abnormal divide’, which exists elsewhere in the world. The findings here support this contention. However, even in this context with their own supportive midwife, the women’s responses suggest that there are limits to the level of influence the women’s midwives can have in the multi-disciplinary tertiary hospital setting. Despite their best efforts they cannot necessarily protect women from poor communication with hospital staff or a sense of loss of control, whether women are transferring or receiving all their care in the hospital setting. While midwives can ‘help bridge the divide’ they alone cannot eliminate it–they are still only one part in a complex system.

The themes identified here are not unique to the context of transfer during childbirth, rather they reflect the key dimensions of ‘patient-centred care’: control, participation in decision-making, support and information [5]. While key to positive birth experiences generally, these constructs have also been linked to birthplace, with women who plan PMU births experiencing greater levels of “support, participation in decision-making, attentiveness to psychological needs and wishes for birth [by midwives], information, and for women feeling listened to” [5].

The study is potentially limited by the small sample size and the sole use of survey data, collected postnatally. The limitations of Likert scale survey responses in this context have been identified previously [15]. None of the focus group participants in the larger study experienced transfer between facilities for the birth of the baby, consequently only survey data were analysed. The study was undertaken in one region of New Zealand, with a sample biased towards those with a moderate ability to read and write English and non-Māori, and damaging earthquakes occurred during the study period, all of which have the potential to affect the findings and outcomes. Self selection bias is also present in the larger study’s cohorts, as all of the women chose their preferred birthplace, leaving open the possibility of psychological or motivational differences between them. Its strengths include the large sample size, high response rate and the comprehensive nature of women’s responses.

## Conclusions

This paper found that, in the context of universal continuity of midwifery care, when the qualitative and quantitative responses were integrated, women understood the potential for plan change or transfer and appeared to be more unhappy about the *decision* to change birthplace plan or transfer than the *transfer* itself. The women indicated that while ‘not to plan’ the experience was okay, if they perceived a sense of control and had effective communication with caregivers and support and information from their midwife. The study is limited by a small sample size and use of only survey data and care must be taken not to generalise the findings from this study. Further research into the views and experiences of women who plan a primary unit birth, but have to change their planned place of birth at any time during the process, will provide a better understanding of this process; and help shape the organisation of maternity systems to better meet the needs of childbearing women.

The implications for practice are that the experience of having to change birthplace plans or transfer can be influenced by caregivers. Those caregivers who enable women to maintain a sense of control, communicate respectfully and effectively with women, and provide support and information to them may facilitate a more positive experience of transfer for all concerned. Continuity of midwifery care was also important for participants. New Zealand has a unique maternity system where all women are offered a model of continuity of midwifery care regardless of where they plan to give birth. The study showed that this model appeared to mitigate the negative aspects of women’s experience of transfer and facilitate positive birth experiences in the face of having to change a planned place of birth.
